# COGNITION, SELF-RATED COGNITIVE HEALTH, AND CONCERNS ABOUT ALZHEIMER’S DISEASE IN OLDER KOREAN AMERICANS

**DOI:** 10.1093/geroni/igz038.988

**Published:** 2019-11-08

**Authors:** Yuri Jang, Nan Sook Park, David Chiriboga, Hyunwoo Yoon, Min-Kyoung Rhee

**Affiliations:** 1 University of Southern California, Los Angeles, California, United States; 2 University of South Florida, Tampa, Florida, United States; 3 Texas State University, San Marcos, Texas, United States

## Abstract

Responding to the dearth of research on cognitive health in older ethnic minorities, the present study explored the associations among cognitive performance, self-rated cognitive health, and concerns about Alzheimer’s Disease (AD) in older Korean Americans. We hypothesized that (1) cognitive performance and self-rated cognitive health would be moderately associated; (2) both cognitive performance and self-rated cognitive health would be associated with concerns about AD; and (3) the effect of cognitive performance on concerns about AD would be mediated by self-rated cognitive health. Analyses of the survey data of 2,150 older Korean Americans (mean age = 73.2) residing in five states provided support for the hypotheses. Cognitive performance, measured with a Korean version of the Mini-Mental State Exam (MMSE), and a single item asking respondents to rate their overall cognitive status on a 5-point scale (excellent/very good/good/fair/poor) were moderately associated. Both poor performance in the MMSE and negative ratings of cognitive health status were associated with increased concerns about AD. Mediation analyses using the macro PROCESS showed that the indirect effect of cognitive performance on the concerns about AD through self-rated cognitive health (−.01 [.002]) was significant (bias corrected 95% confidence interval for the indirect effect = −.02, −.01). That is, the effect of cognitive performance on the concerns about AD was mediated by individuals’ subjective evaluations of their cognitive health. Findings not only help better understand the psychological mechanisms that underlie cognitive health and AD concerns but also suggest avenues for interventions.

